# Vortex Shedding Optical Flowmeter based on Photonic Crystal Fiber

**DOI:** 10.1038/s41598-019-40464-2

**Published:** 2019-06-05

**Authors:** Venugopal Arumuru, Jitendra Narayan Dash, Dhrubaraj Dora, Rajan Jha

**Affiliations:** 1 Applied Fluids Group, School of Mechanical Sciences, IIT Bhubaneswar, Khurda, 752050 India; 2 School of Physical Sciences, NISER Bhubaneswar, Khurda, 752050 India; 3 Nanophotonics and Plasmonics Laboratory, School of Basic Sciences, IIT Bhubaneswar, Khurda, 752050 India

**Keywords:** Mechanical engineering, Optical sensors, Imaging and sensing

## Abstract

In the present work we propose a PCF (photonic crystal fiber) based Modal interferometer detector for sensing low flow velocity by detecting the frequency of vortices shed from a bluff body. The proposed novel design encapsulates the interferometric arm inside a metal casing to protect the sensor from harsh process fluids. The characterization of the developed probe is conducted under no flow conditions using a piezo actuator to evaluate the sensor response over wide frequency range (0–500 Hz). The developed sensors shows a reasonably flat response in the tested frequency range. Experiments are conducted by employing the developed sensor behind a bluff body of a vortex flowmeter to measure the frequency of the shed vortices and hence, the fluid flow rate. The low flow rate sensitivity of the vortex flowmeter is improved many folds by using the present sensor and the minimum Reynolds number detected is *Re* = 5000. A linear trend is observed between the frequency of the vortices and the flow velocity which is desirable for fluid flow measurement. The PCF based interferometric sensor with metal encapsulation makes the vortex flowmeter, sensitive at low flow rates, robust and economical to be used in industrial application.

## Introduction

Flow measurement has a wide range of significant applications in various industrial, chemical and biological fields. Various kinds of new technology flow meters which include Coriolis, ultrasonic and electromagnetic flow meters have been developed and reported to date^1^. Although Coriolis and ultrasonic flow meters offer features such as high accuracy and turndown ratio, they are bulky and not cost effective. These flowmeters are mainly preferred for custody transfer application, which demands high accuracy. The major advantage of Coriolis flowmeter is direct measurement of mass flow rate. Apart from cost, pressure drop is another drawback of Coriolis flowmeter. On the other hand, electromagnetic flow meters are economical but their utility is limited to conducting fluids (liquids). However, for industrial applications, vortex shedding based flow meters are a better choice due to their ability for metering air, steam as well as corrosive fluids at reasonably lower price. The absence of rotating parts and operation over a wide range of Reynolds number is the unique features of vortex flow meters^2^. However, some of the operational issues like sensitivity to external vibrations especially at low flow rates are hindering their usage in harsh conditions. Another deterrent in their market popularity is their sensitivity and accuracy at low flow rates. Numerous reviews on vortex flowmeters design such as by Venugopal *et al*.^2^ and Pankanin^3^ have been reported in the literature. The sensitivity to external vibrations and low flow rate issues have been addressed mainly by proposing design modifications in the sensor, signal processing and modifications in the shape of the bluff body^4–11^. The acceptance of vortex flowmeter is also limited to low temperature applications, due to the operational temperature limits posed by piezo and capacitive type sensors. These sensors are functional only up to a maximum temperature limit of 300 °C limited by their Curie temperature. However, a wide range of industrial applications encounter fluid flow at high temperature and these includes, thermal power plants, chemical & process industries to name a few. Further, in oil industry, the flow measurement has to be accomplished for inflammable liquids in harsh environment conditions. Conventional vortex flowmeters are not preferred for the above applications. Hence, there is a need for the development of a sensor for vortex flowmeter which is reliable at high temperatures, immune to corrosion, chemical reactions and electromagnetic interference.

The above conditions can be suitably achieved by employing optical fiber based sensor which are not only compact but also can be utilized for remote and online sensing based applications. Several procedures have been followed to measure flow rate using conventional as well as specialty optical fibers. For example, a non-adiabatic tapered single mode fiber (SMF) has been utilized to monitor the air flow by measuring the phase shift due to pressurized air flow^12^. Further, a core offset Fiber Bragg Grating (FBG) has been utilized to monitor thermal gas flow where the shift in Bragg peak occurs due to the variation in temperature^13^. However, both the above sensors have been designed for gas flow (clean fluid) measurement at ambient temperatures and not tested for liquid flow measurement. The flow rate of liquids have also been measured using fiber based devices. For example, a fiber based opto-fluidic flow meter has been reported to have a sensitivity of the order of 10 nl/min. However, in this complicated experimental setup, a micro particle has to be optically trapped and the change in balance of the micro particle with the flow of liquid has to be utilized to detect the flow rate^14^. Further, a macrobend fiber has also been utilized for flow sensing by varying its curvature with flow of liquid^15^. Moreover, a cobalt doped fiber in combination with FBGs has been utilized for micro fluidic flow meter where the doped fiber has been heated with laser and the peak shift due to the variation in temperature has been considered for detection of flow rate^16^. Moreover, FBGs have also been proposed for vortex based flowmeter application^17,18^. However, the major drawback of this technique is fabrication complexity, sensitivity to temperature change, and poor sensitivity at low flowrates.

In addition to the above designs, optical fibers have also been utilized for vortex flow sensing. Most of these works are based on multi-mode fiber (MMF). For example, a multimode fiber kept in a copper tube along with the fluid and the flow of fluid causes the fiber to move thereby resulting in phase deviation in optical path along the fiber^19^. Further, MMF mounted on bluff body has been subjected to transverse force caused by the vortex and the corresponding bending loss of fiber has been considered to monitor the vortex shedding frequency^20^. In another set up, the MMF has been placed perpendicular to the flow and the generated vertices around each side of the fiber exerts force on the fiber. This type of flow meter has been used for water speed of 5 m/sec^21^. Further, highly multimode fibers with core diameter of 200 and 300 µm have been used to study the frequency detection using photo detector^22^. However, most of these sensors are based on loss in output intensity and the performance of these sensors is vulnerable to fluctuation in source power and hence the stability and reproducibility of the device performance is a concern. In addition to this, fiber based interferometers have also been utilized for flow sensing applications. In this technique, the vortex frequency is monitored by the vibration of the interferometer caused due to the vortex and therefore one needs to monitor the vibration of the interferometer and the corresponding shift in interference peak. For example, vibration sensor based on multi core fiber interferometer has been reported^23^. However, the fabrication of such customized and costly fiber is difficult and is not easily available commercially. Further it has doped core which may be vulnerable to high temperature applications. Also, it is worth noting that this sensor use was restricted to only vibration measurement. Therefore, in this paper, we propose and demonstrate a novel Photonic Crystal Fiber (PCF) based interferometer for detection of vortex shedding frequency at low flow rates and hence improves the overall operating range of the flow meter. The basic principle of the sensor is based on the vibration of the interferometer which causes the bending of the interferometer thereby resulting in shift of the interference peak. The sensor is compact and simple to fabricate as it requires splicing of SMF on both sides of a section of PCF. Such type of sensor can be operated in harsh environmental conditions as they are non-corrosive and made up pure silica having high melting point and hence can be operated in high temperature fluidic environment^24^. Further, the zero downtime of the industrial plant is another advantage of the proposed device as the damaged sensors can be replaced swiftly and easily due to the simple splicing process without much cost escalation.

## Method

### Fabrication of the Probe

**Figure 1:**
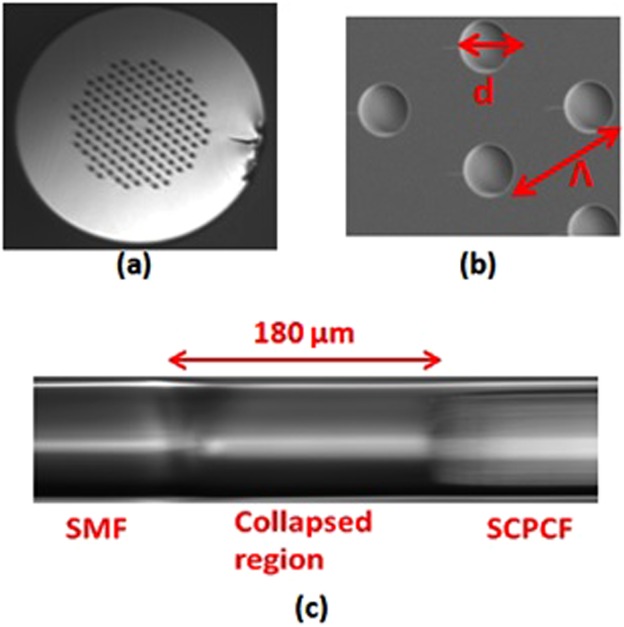
(**a**) Shows the microscopic picture of PCF, (**b**) shows the zoomed view of holes of PCF and (**c**) shows the microscopic picture of the collapsed region at SMF-PCF junction.

**Figure 2:**
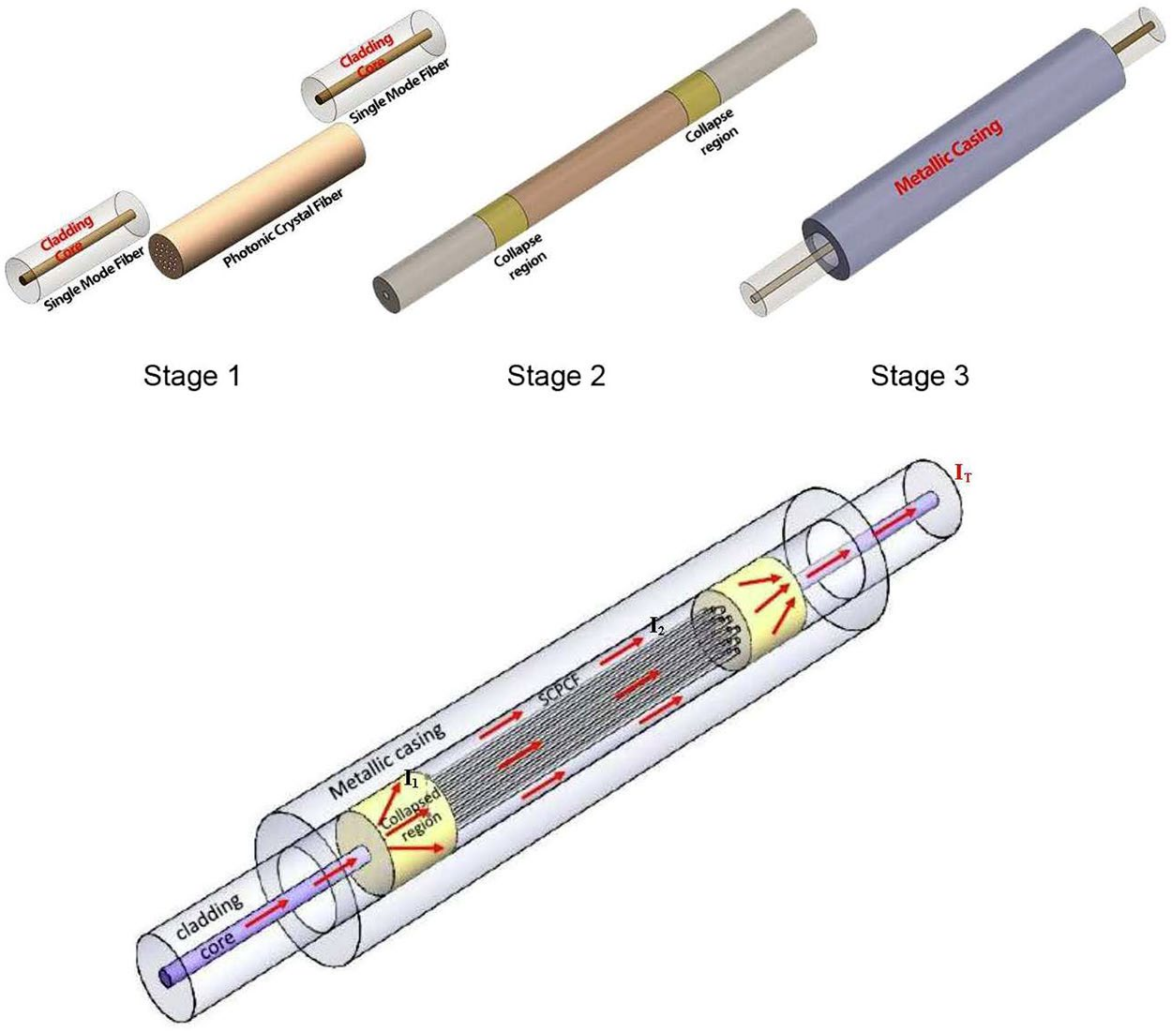
Fabrication and assembly stages of the sensor.

The basic procedure for the fabrication of probe involves splicing of SMF and solid core PCF. The SMF considered for this has a core diameter of 8.5 *μ*m and cladding diameter of 125 *μ*m. On the other hand, the PCF (LMA 8) has a core diameter of d = 8.5 *μ*m while the diameter of cross section is 125 *μ*m as shown in Fig. 1(a). The average distance between two consecutive holes i.e pitch is Λ = 5.6 *μ*m and the average diameter of holes is 2.32 μm as shown in Fig. 1(b). The fabrication of probe is accomplished by following the steps as follows. First, the outer coating of a section of SCPCF (LMA 8) is stripped and cleaned using a stripper and acetone respectively. The cleaned SCPCF and SMF is cleaved using a cleaver. The cleaved section of one end of PCF is spliced with SMF. The splicing leads to the collapse of holes of PCF over a length of 180 µm as shown in Fig. 1(c). Following the same procedure, the other end of the PCF is spliced with another section of SMF resulting in SMF-PCF-SMF (SPS) configuration. The step by step process for the device fabrication is shown in Fig. 2. Here excitation and recombination of modes is carried out with permanent and stable splices which does not degrade with time and temperature. However, it is to be noted that the length of the collapse region depends upon the arc power and arc time of the fusion splicer. These parameters can be precisely tuned to control the length of the collapsed region. So the method is highly reliable and repeatable. The outer cladding is further encapsulated inside a hollow steel tube of 200 *μ*m, the annular region is filled with glue to ensure mechanical coupling between the interferometric sensor and the metal casing to effectively transfer vibrational energy from the metal casing to the sensor as shown in in Fig. 2. The position of the glue has been optimized and is placed in such a way that the cladding modes of PCF do not get affected so that the interference pattern is well retained.

### Probe characterization

**Figure 3:**
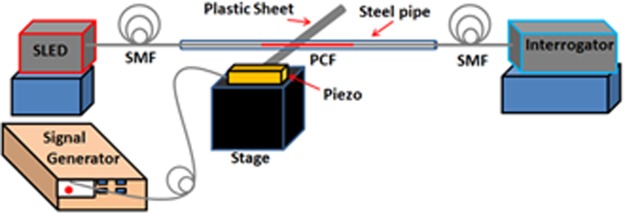
Experimental set up for piezo based vibration sensing.

Before utilizing the probe for flow measurement, the response of the probe to the input vibrational frequency is first studied using a piezo-actuator. This is due to the fact that the presence of a bluff body in the pipe (in which flow velocity is to be measured) results in the generation of vortex shedding frequency and the response of the probe to this frequency is to be measured. Further, the objective of this experiment is to evaluate the response of the optical probe for wide range of frequency. Since, the amplitude and frequency of vortex shedding is unknown a prior, hence the tests are conducted with a piezo actuator, which is excited at a known frequency and amplitude. This test is necessary to characterize the probe for wide range of frequency and amplitudes of vibrations before using it in an unknown environment. The two ends of the probe are fixed between two stationary stages and a plastic sheet with adequate stiffness is attached to the piezo as shown in Fig. 3. The length of the probe is optimized and fixed at 40 mm. The plastic sheet is kept just below the mid part of the probe i.e. below the mid part of PCF as shown in Fig. 3. The piezo is connected to a signal generator that can be operated at different amplitude and frequencies. The probe is subjected to different amplitudes and frequencies through the piezo-actuator to evaluate the response of the probe. The data is acquired at a sampling rate of 1 kHz.

**Figure 4:**
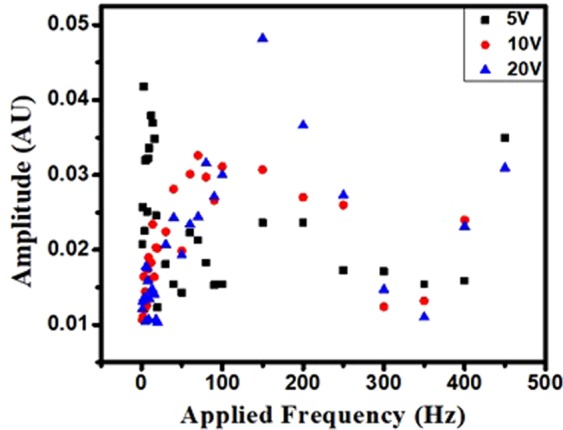
Variation in amplitude of a peak with applied frequency for different values of input amplitude for a given PCF length of 40 mm.

The response of the probe to different input amplitudes is studied by varying the input voltage from 5 V to 20 V. As can be seen from the Fig. 4, the variation in amplitude to applied frequency is almost uniform. This flat response is advantageous in the present application as the flow rate is inferred from the frequency shift and the present technique is not based on amplitude tracking. It is to be noted that the tests are conducted with a resolution of 1 Hz in the range of 0–20 Hz, with a resolution of 5 Hz in the range of 20–50 Hz and with 10 Hz resolution in the range of 50–500 Hz. Since, nearly 20 data points are obtained in the range of 0–20 Hz, hence the density of the data points is more near 0 Hz in Fig. 4. Here, the change in vibrational amplitude of the peak can be neglected due to the variation in second decimal. Further, large variation in amplitude may lead to error in tracking of interference peaks. This is due to the fact that in order to track a given peak of the interference pattern, a particular amplitude limit is fixed in IMON (interrogator). If the change in amplitude becomes very high, then other peaks may come into picture and this may lead to misleading readings and hence negatively impact the reliability of the measurements. From the above discussions, it can be concluded that the change in amplitude of peak corresponding to applied frequency is insignificant. Therefore, we proceed to use this probe for flow sensing based on wavelength interrogation.

### Flow meter operational principle

**Figure 5:**
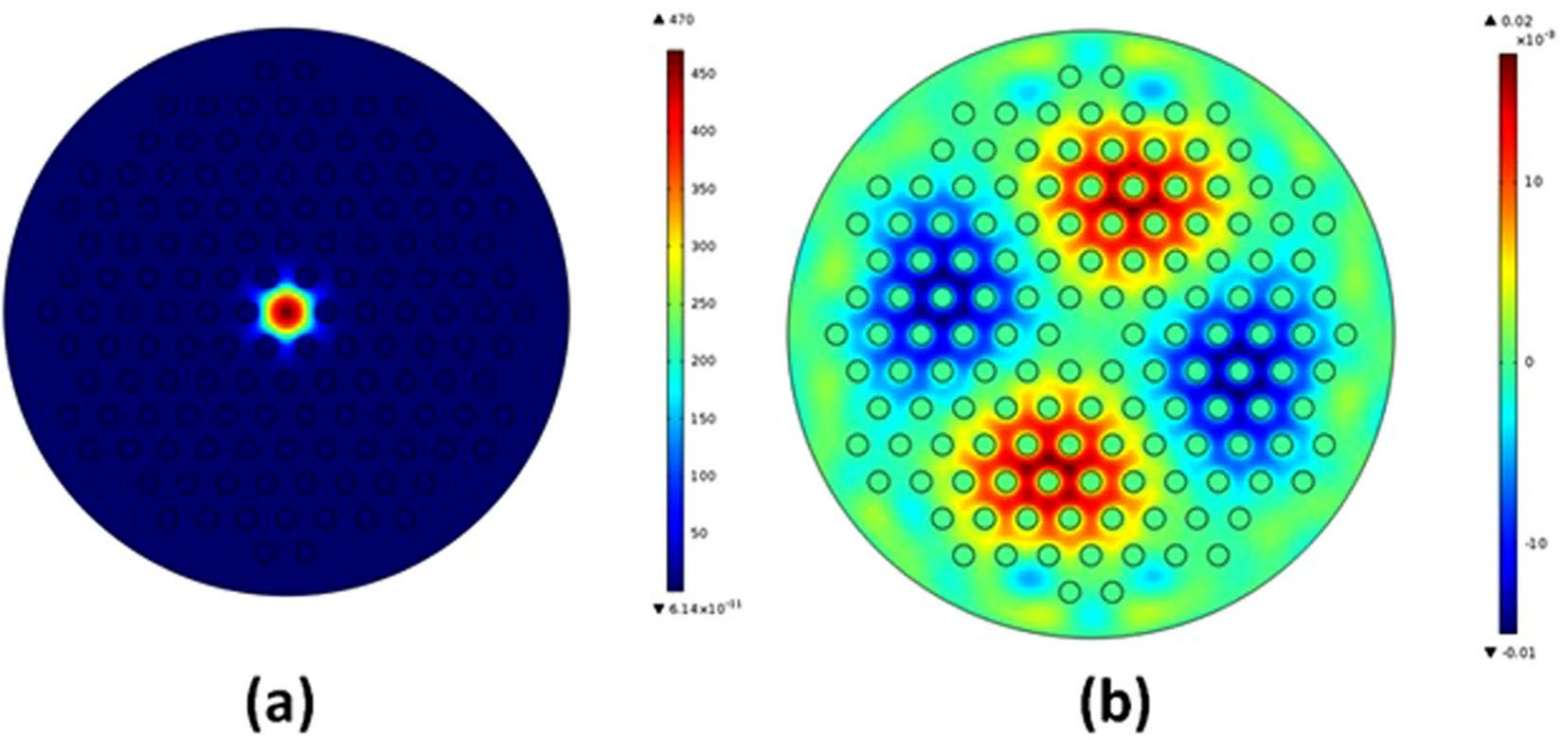
Simulated core (LP_01_) and cladding (LP_21_) mode of PCF.

Light is launched into the probe from a broadband SLED (super luminescent light emitting diode) having a peak wavelength at 1550 nm and spectral width of 35 nm. The use of SLED is advantageous considering its high power and low divergence. This is due to the fact that the fabrication of the probe involves splicing of different types of fibers (SMF-PCF) and the splicing junction of the fibers lead to more losses. The output is analyzed using an interrogator (IMON) which acted as a spectrometer. When light propagates from SMF to PCF, the fundamental mode of SMF broadens upon reaching the collapsed region at the junction of these two fibers as shown in Fig. 2. The broadening of the mode results in the excitation of core and cladding modes in PCF as shown in Fig. 5. These modes again combine at the second collapsed region at the other end of PCF and subsequently filtered through the core of SMF. Due to the different propagation constants, the core and cladding mode accumulate phase difference and this leads to the formation of interference pattern upon combination of these modes. Considering the intensity of core and cladding modes as *I*_1_ and *I*_2_, the resultant intensity *I*_*T*_ of the probe can be written as

1 $${I}_{T}={I}_{1}+{I}_{2}+2\sqrt{{I}_{1}{I}_{2}}\,\cos (\frac{2\pi {\rm{\Delta }}{n}_{eff}L}{\lambda })$$

Any external perturbation that affects the effective index of either the core mode or the cladding mode, will ultimately affect the resulting interference pattern as can be realized from Equation(1). In our case, the vibration of piezo leads to the vibration of the plastic sheet which in turn vibrates the PCF. This results in the formation of a microbend and subsequent shift in the wavelength of the peak of the interference pattern. The shift in interference pattern may be attributed to the shift in cladding mode of the PCF towards outer region and subsequent change in effective index of cladding mode.

### Experimental Set up

**Figure 6:**
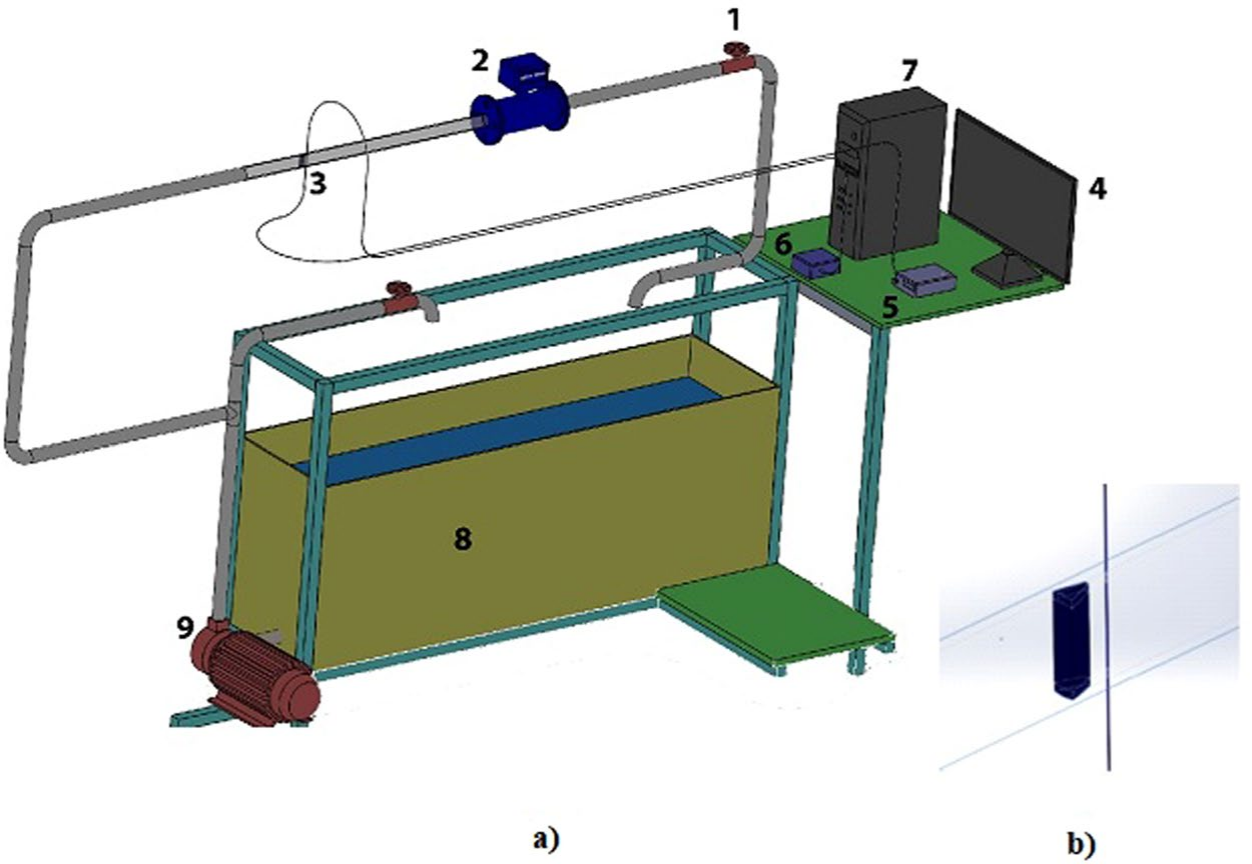
(**a**) Experimental set up for detection of flow of water in pipe. The numbers refer to various parts of the experiment as follows. (1) Ball valve (2) Electro-magnetic vortex flowmeter (3) SMF-PCF-SMF optical fiber (4) Desktop (5) Super-luminescent Diode source (6) I-MON (7) CPU (8) Water tank (9) Centrifugal pump (0.5HP). (**b**) Shows the trapezoidal bluff body.

**Figure 7:**
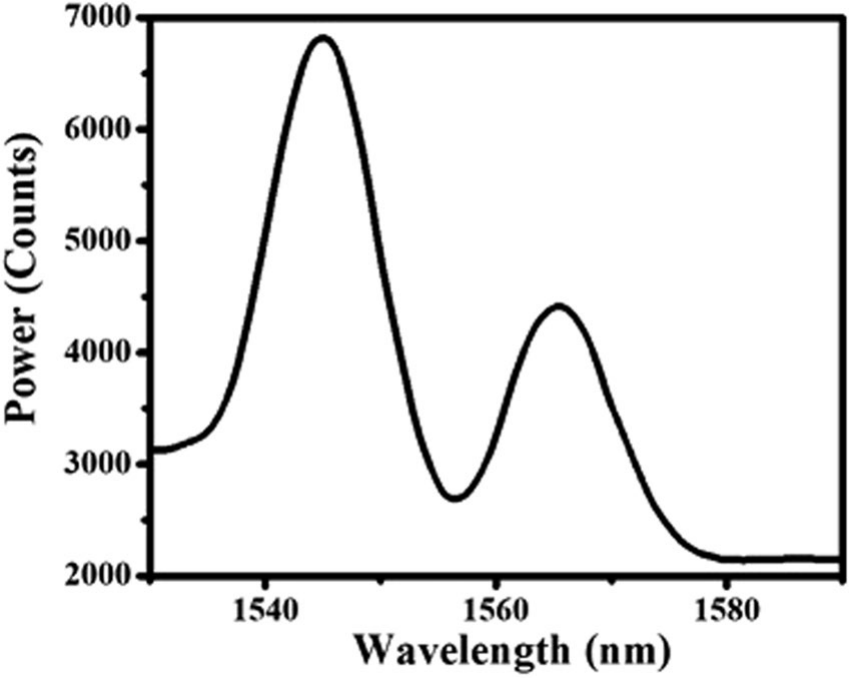
Interference pattern corresponding to wavelengths of 1545.01 nm and 1565.51 nm.

The experimental setup for the flow measurement is shown in Fig. 6(a). As can be seen from the figure, a centrifugal pump (0.5Hp) is connected to the pipe through which flow of water is established. The inner diameter of the pipe is 22.45 mm. In order to regulate the flow of water, a gate valve is connected to the main line (section of pipe through which flow is to be monitored). Further, the mechanical vibration caused due to the pump is nullified using dampers on both upstream and downstream of the main line. A trapezoidal bluff body with blockage ratio of 0.28 is connected along the main line as shown in Fig. 6(b). The probe (SMF-PCF-SMF) is embedded in a micro steel pipe with inner and outer diameter of 200 µm and 400 µm respectively. The probe kept in the steel pipe is attached to the pipe vertically as shown in Fig. 6(a). The length of PCF considered for this is nearly equal to the inner diameter of pipe (22.45 mm) through which water flows. One end of the probe is connected to SLED and the other to the interrogator. The resulting interference pattern of the probe is shown in Fig. 7. As evident from the figure, there are two peaks corresponding to wavelengths of 1545.01 nm and 1565.51 nm.

### Reynolds number

Usually, the flow condition of fluids or the type of fluid flow can be predicted using a parameter called Reynolds number and this can be defined as:

2 $$Re\,=\frac{\rho \,\times V\times d}{\mu }$$

where, the terms *ρ*, *D* and *μ* refer to the density of water, diameter of pipe and dynamic viscosity of water. As the density of water is a function of temperature, we have considered the density as 998 Kg/m^3^ considering the room temperature as 20 °C. Further, for the same condition, the dynamic viscosity of water is 0.001 × 10^−3^ Kg/m.s. Therefore, the variation in volume flow rate affects the flow velocity which in turn changes the Reynolds number. In order to see the variation in Reynolds number with velocity of flow, we varied the velocity from 0.23 m/sec to 2 m/sec and the corresponding Reynolds number is found to vary from 5000 to 45000.

Several industrial applications demands metering of the flow at low Reynolds number, presently convention vortex flowmeter use a reduced bore design, which are capable of measuring low Reynolds number flows. However, the major disadvantage of such design is permanent pressure loss, which impose financial burden and hence, are not preferable in industrial application. Hence, the present design has significant merit as the design is capable of measuring low Reynolds number without addition pressure loss and cost escalation. It is interesting to note that flow measurement using vortex flow meters at high Reynolds number is relatively easier since the signal strengths are sufficiently high. Hence, in the present study the focus is restricted to measure low Reynolds numbers.

Further, when the water flows through the pipe, the trapezoidal bluff body acts as an obstacle and as a consequence of this vortices are created downstream of the bluff body. These vortices shed at a fixed rate (*f*), which is directly proposal to the velocity of the flow.

### Strouhal number

The non-dimensional vortex shedding frequency is a function of flow velocity (*V*), width of bluff body (*d*), which is termed as Strouhal number and is defined as:

3 $$St=\frac{fd}{V}$$

**Figure 8:**
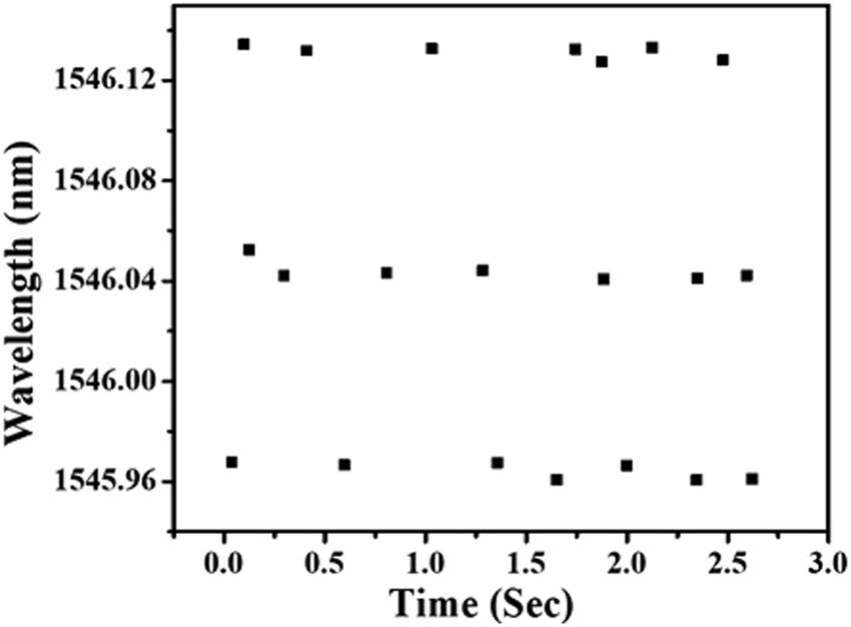
Peaks corresponding to wavelengths of 1545.01 nm and 1565.51 nm.

Where, the Strouhal number describes the dynamics of oscillating fluid flow. The alternately shed vortices from the bluff body impinges on the sensor placed downstream of the bluff body as shown in Fig. 6(b). This vortex shedding frequency is coupled to the steel pipe and subsequently it causes vibrations in the probe. As the PCF section of the probe is attached to the pipe, this vibration induces a dynamic curvature on it. The curvature in PCF leads to the change in effective index of cladding mode which in turn changes Δn_eff_ and this causes the interference pattern to shift. Although there are two peaks in the interference pattern, we fixed the power limits of IMON in such a way that only one peak (1545 nm) is tracked. The vortex shedding frequency is reflected in the variation in wavelength of the interference peak due to the bending of PCF. As can be seen from Fig. 8, maximum variation of wavelength of the peak with time occurs between 1545.95 nm to 1546.13 nm for flow velocity of 0.45 m/sec i.e there is a shift of 180 pm in wavelength of the interference peak corresponding to the vibrations generated due to the creation of vortex. It is to be noted that the peak wavelength gets negligibly affected by temperature. This is due to the fact that the air holes have negligible thermo-optic coefficient. Further, the absence of doping in the core of PCF also leads to negligible shift in interference peak^24–27^.

**Figure 9:**
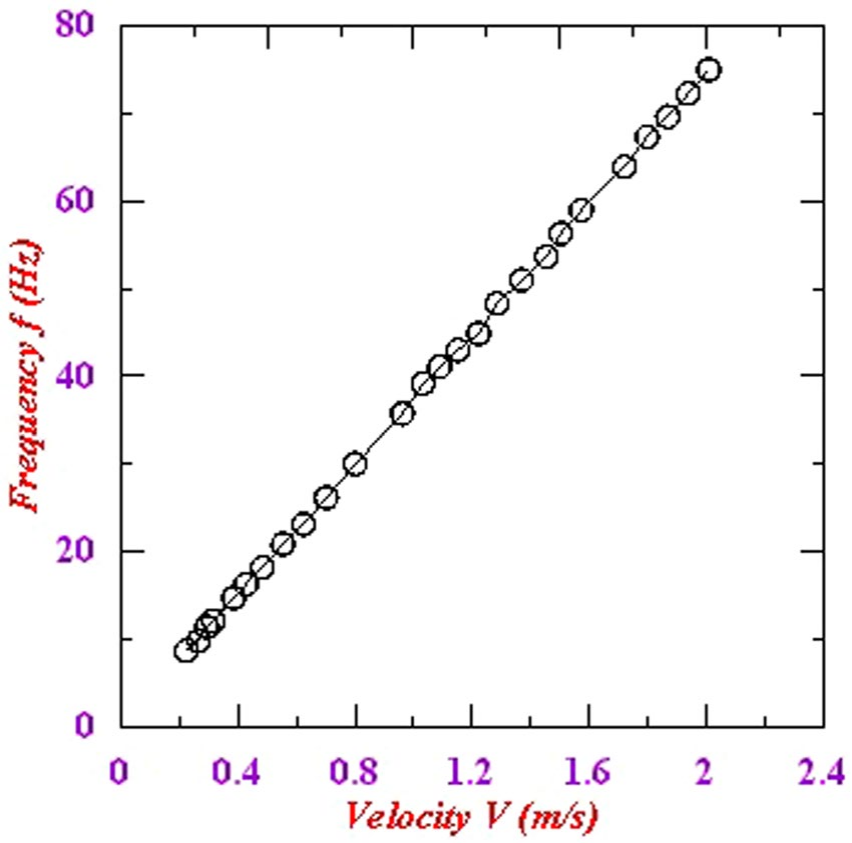
Variation in frequency of the interference peak with flow velocity.

**Figure 10:**
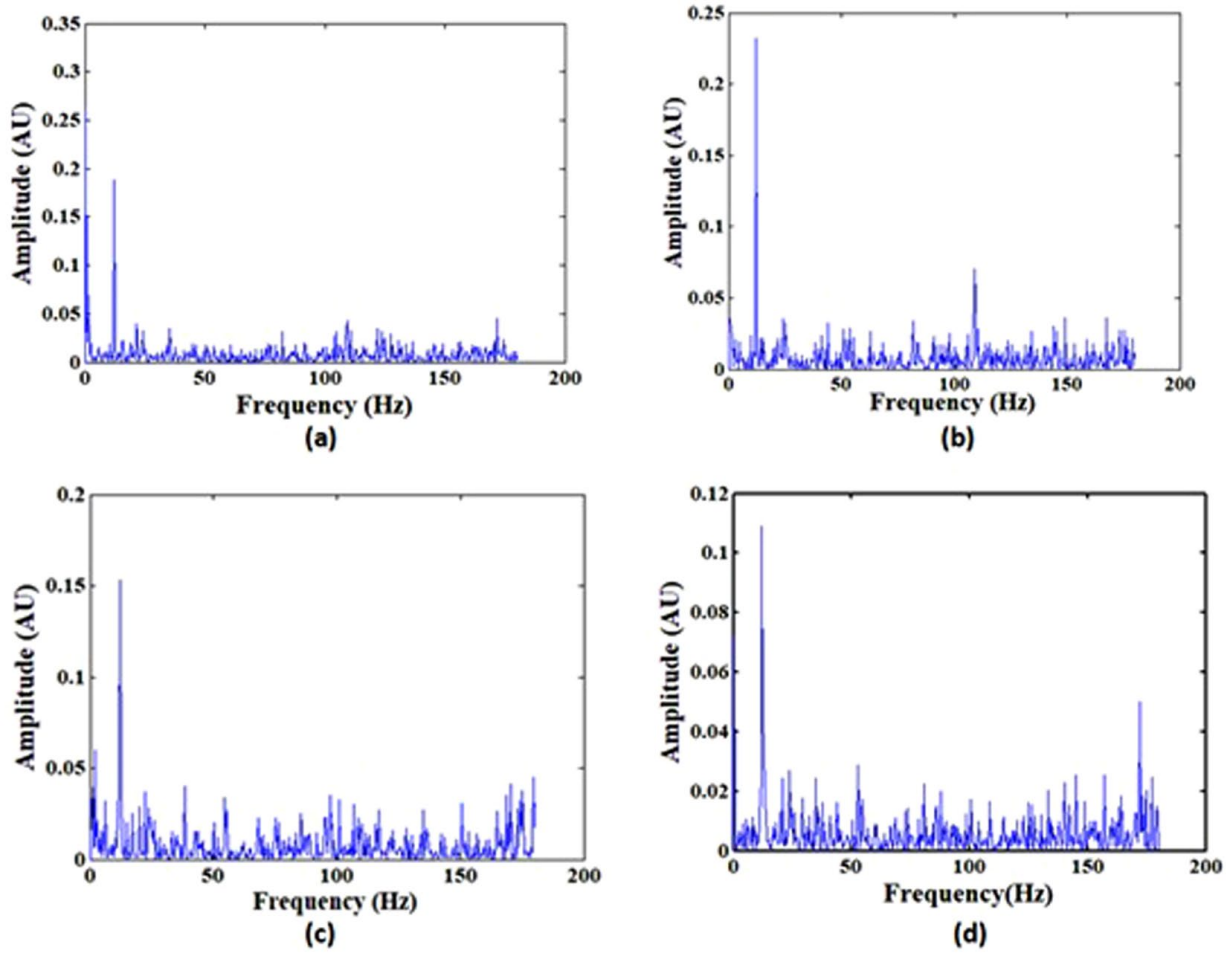
FFT of the variation in frequency of the interference peak for a given flow velocity (0.31 m/sec).

The corresponding FFT of the variation of this wavelength is calculated for each flow velocity. The variation in frequency with flow velocity is plotted in Fig. 9. As can be seen from the figure, this variation shows linear behavior. Further, for a given flow velocity, we collected data four times and plotted the FFT in Fig. 10 for better averaging. As evident from the figure, the spatial frequency for each measurement corresponds to 12.16 Hz which shows the consistency of the measurement and hence good repeatability in the measurement.

**Figure 11:**
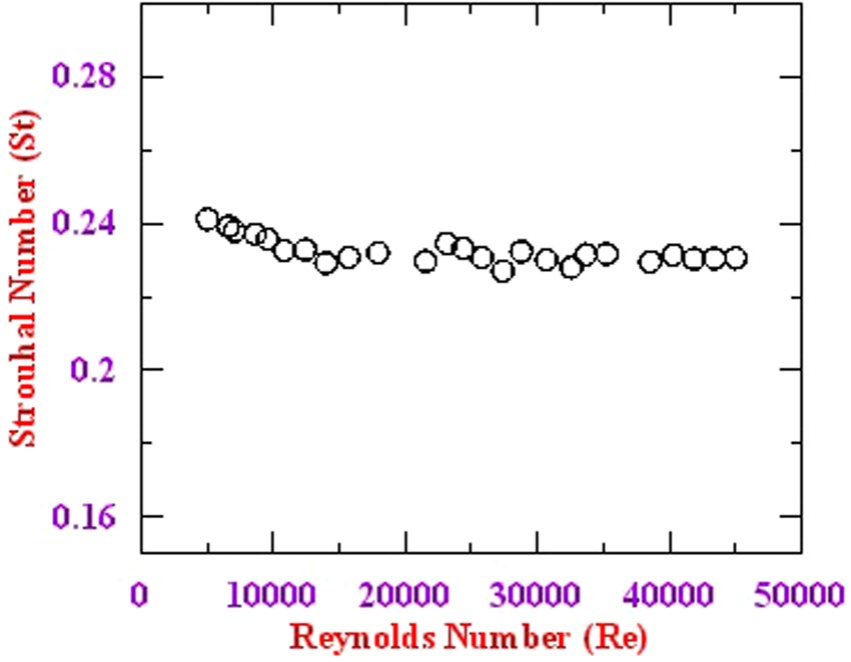
Variation in Strouhal number with Reynold’s number.

In addition to the above measurement, the variation of Strouhal number with change in Reynolds number is shown in Fig. 11. As can be seen from the figure, the variation occurs in the second decimal range and therefore it can be considered as insignificant variation. Hence, the velocity of the flow can be computed by just measuring the frequency of the shed vortices in real application, which is the principle of operation of vortex flowmeter. The experiments have been repeated in the laboratory for prolonged duration and the results are found to be consistent. In the present study, we have measured a minimum Reynolds number Re = 5000, this lower limit can be further extended by improving the sensitivity of the probe. However, the present experimental setup doesn’t allow to achieve stable low flow velocities.

## Conclusion

The present study successfully demonstrate a novel highly sensitive senor for detecting low flow velocities using PCF based model interferometer. The response of the probe is observed to be flat for a wide range of frequencies and hence enables frequency detection based on wave length shift. The developed sensor is tested with vortex flowmeter in laboratory and the minimum Reynolds number measured is 5000, which is significantly lower compared to conventional vortex flowmeters employing Piezo and capacitive type sensors. A linear trend is observed between the frequency of the shed vortices and flow velocity and hence the Strouhal number is observed to be nearly constant in the range of Reynolds number tested in the present study (Re = 5,000–50, 000). The encapsulated design of the present sensor makes it suitable for direct industrial applications where high temperatures and harsh process fluids are encountered.
